# Risk factors for *Streptococcus suis* infection: A systematic review and meta-analysis

**DOI:** 10.1038/s41598-018-31598-w

**Published:** 2018-09-06

**Authors:** Ajaree Rayanakorn, Bey-Hing Goh, Learn-Han Lee, Tahir Mehmood Khan, Surasak Saokaew

**Affiliations:** 1 Novel Bacteria and Drug Discovery Research Group (NBDD) & Biofunctional Molecule Exploratory Research Group (BMEX), Biomedicine Research Advancement Centre (BRAC), School of Pharmacy, Monash University Malaysia, Bandar Sunway, Selangor Darul Ehsan Malaysia; 2 Center of Health Outcomes Research and Therapeutic Safety (COHORTS), School of Pharmaceutical Sciences, University of Phayao, Phayao, Thailand; 3 Center of Pharmaceutical Outcomes Research (CPOR), Faculty of Pharmaceutical Sciences, Naresuan University, Phitsanulok, Thailand; 4 The Institute of Pharmaceutical Sciences (IPS), University of Veterinary & Animal Sciences (UVAS), Outfall road, Lahore, Pakistan

## Abstract

*Streptococcus suis* (*S*. *suis*) is a gram-positive bacterial pathogen in pigs which can cause serious infections in human including meningitis, and septicaemia resulting in serious complications. There were discrepancies between different data and little is known concerning associated risk factors of *S*. *suis*. A systematic review and meta-analysis was conducted to investigate on S. *suis* infection risk factors in human. We searched eight relevant databases using the MeSH terms “Streptococcus suis” OR “Streptococcus suis AND infection” limited in human with no time nor language restriction. Out of 4,999 articles identified, 32 and 3 studies were included for systematic review and meta-analysis respectively with a total of 1,454 *Streptococcus suis* cases reported. *S*. *suis* patients were generally adult males and the elderly. The mean age ranged between 37 to 63 years. Meningitis was the most common clinical manifestation, and deafness was the most common sequelae found among survivors followed by vestibular dysfunction. Infective endocarditis was also noted as among the most common clinical presentations associated with high mortality rate in a few studies. Meta-analyses categorized by type of control groups (community control, and non-*S*. *suis* sepsis) were done among 850 participants in 3 studies. The combined odd ratios for studies using community control groups and non-*S*. *Suis* sepsis as controls respectively were 4.63 (95% CI 2.94–7.29) and 78.00 (95% CI 10.38–585.87) for raw pork consumption, 4.01 (95% CI 2.61–6.15) and 3.03 (95% CI 1.61–5.68) for exposure to pigs or pork, 11.47, (95% CI 5.68–23.14) and 3.07 (95% CI 1.81–5.18) for pig-related occupation and 3.56 (95% CI 2.18–5.80) and 5.84 (95% CI 2.76–12.36) for male sex. The results were found to be significantly associated with *S*. *suis* infection and there was non-significant heterogeneity. History of skin injury and underlying diseases were noted only a small percentage in most studies. Setting up an effective screening protocol and public health interventions would be effective to enhance understanding about the disease.

## Introduction

*Streptococcus suis* (*S*. *suis*) is a gram-positive bacterial pathogen in pigs which can cause serious infections in human including meningitis, septicaemia, and others^1–3^. The number of *S*. *suis* cases has notably increased during the past few years with the highest prevalence rate in Southeast Asia region where there is a high rate of swine consumption^1^. Majority of increased cases are originated from Thailand and Vietnam, making both countries the highest disease prevalence stratum globally^4^.

About two thirds of *S*. *Suis* infected patients developing meningitis syndrome in which deafness and vestibular dysfunction were the most common complications found among survivors^4^. Although the case fatality rate among *S*. *suis* meningitis cases is lower than those caused by other agents^5,6^, the rates of neurological and other sequelae found among *S*. *suis* meningitis survivors seem to be higher than other bacterial meningitis according to a recent meta-analysis^7^. Hearing loss was the most common sequelae found (33.9%), followed by multiple impairments (19.7%) in bacterial meningitis with majority of cases concentrated in the Africa and Southeast Asian regions^7^.

While pig-related occupation is a main risk factor for human *S*. *suis* infection, pig exposure is not present in all cases of *S*. *suis* infection^1^. In Western countries, *S*. *suis* infection normally occurs among certain risk population particularly farmers and abattoir works involving meat processing^8,9^ whereas there were less than 50% of occupational exposure cases documented in Asian countries^10,11^. A lower proportion rates of occupational exposure to pigs were found in Thailand and Vietnam among *S*. *suis* infected patients^4^. This reflects that the risk of infection may be among general population^12^ and other risk factors such as raw or partially cooked pork consumption habit may play an important part of infection in Asia^4^.

Up to date, there have been no systematic reviews that comprehensively investigate on *Streptococcus suis* infection risk factors in human. This systematic review and meta-analysis aims to identify potential risk factors associated with *S*. *suis* infection as well as provides an update on evidences regarding clinical presentations and outcomes of the disease.

## Results

### Study selection

**Figure 1:**
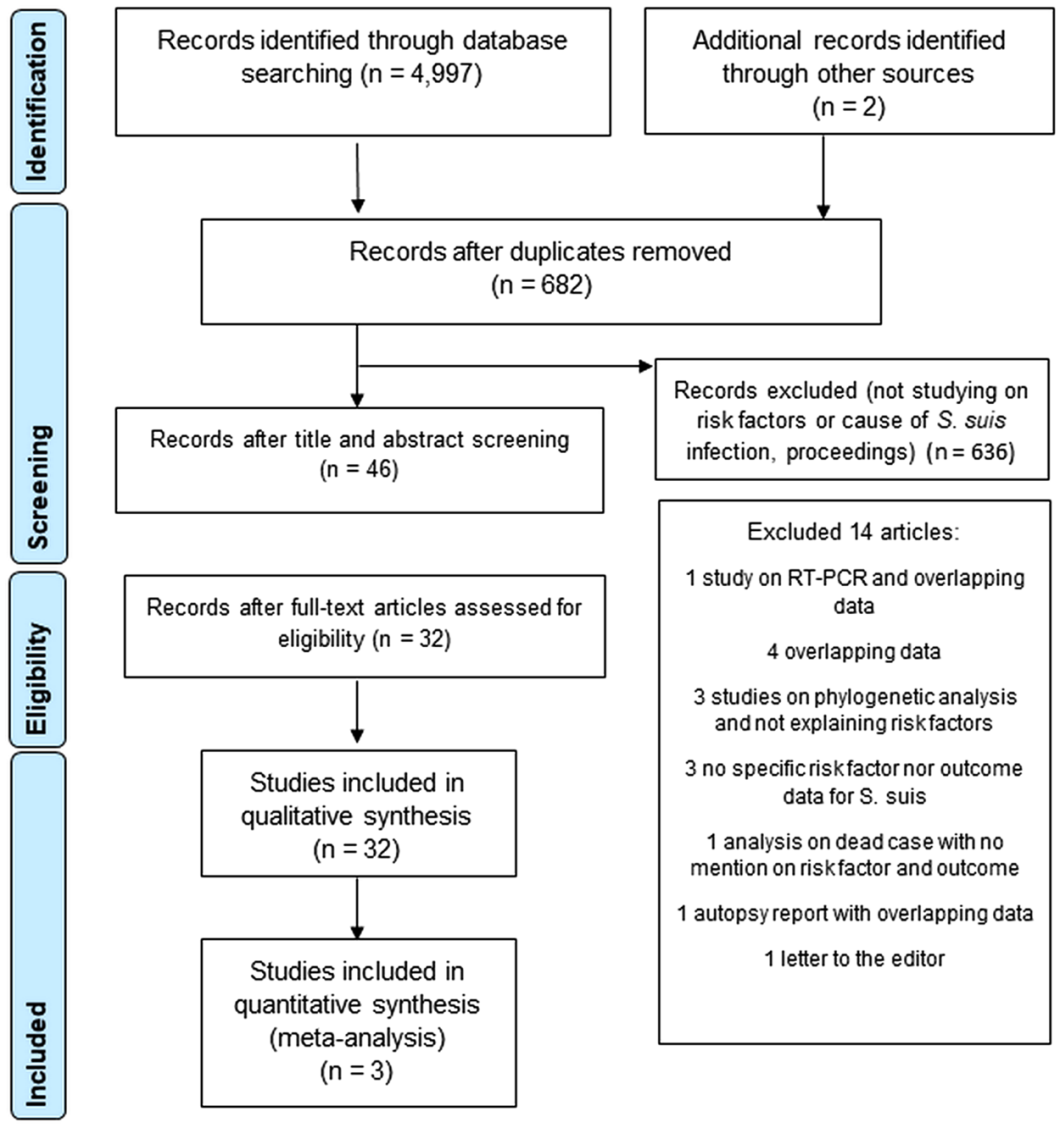
PRISMA flow chart of study selection process.

A total of 4,999 articles were identified in the initial searches from eight databases (n = 4,997) and other sources (n = 2). There were 682 records remaining after removing duplicates in which 636 citations that were proceedings or did not contain risk factors were excluded upon title and abstract screening. There were 32 articles included in systematic review^5,9–11,13–40^ and 3 case-control studies^19,37,38^ in the meta-analysis after full texts evaluation. The PRISMA flow chart describing the study selection process was shown in Fig. 1.

### Study characteristics

The key study characteristics were shown in supplementary appendix Table S1. Included studies with different study designs were conducted in 9 distinctive countries. Among these, there were 1 randomized double-blind, placebo-controlled trial^10^, 3 case-control studies in which there were 1 matched case-control^37^, 1 retrospective case-control^38^ and 1 prospective case-control studies^19^, 28 descriptive studies including 3 public health surveillance studies^27,40,41^, 2 outbreak investigations^36,39^ and 1 epidemiological analysis in China^23^, a population-based study on a food safety campaign^30^ and a retrospective cohort identifying risk factor for *S*. *suis* mortality^34^, 3 retrospective reviews^16,20,21^ and 17 case reports or case series^9,11,13–15,17,18,22,24–26,28,29,31–33,35^. There were 27 articles in English and 5 in other languages; 3 in Chinese^23,24,37^, and 1 each in Croatian^17^ and Thai^40^.

Majorities of studies were from Asia mainly Thailand, China, Hong Kong and Vietnam. Fourteen studies were from Thailand^16,18,20,21,26–31,33,34,39,40^, four each were from China^23,24,36,37^, Hong Kong^11,15,22,25^ and Vietnam^10,19,35,38^, two studies were from the Netherlands^9,13^, and one study each from Japan^14^, Serbia^17^, the UK^32^ and Togo^41^. Two out of the four articles from China were from epidemiological investigation in Sichuan outbreak in 2005^36,37^.

The year that the studies were published varied between 1983 and 2017. The number of patients included in each study ranged from 4 to 215 patients.

### Risk of bias assessment

The results on risk of bias assessment for the three case-control studies using the Newcastle Ottawa Scale (NOS) were in Table 1. The results showed diverse quality among three studies. Based on overall assessment in terms of “selection”, “comparability”, and “ascertainment of exposure”, there was only one study that attained a high score^19^ whereas one study each may be classified as moderate^38^ and low quality^37^.

**Table 1:** Results of critical appraisal of included case-control studies based on the Newcastle-Ottawa Scale (NOS).

Study	SELECTION				COMPARABILITY		EXPOSURE	
	1. Is the case definition adequate?	2. Representative of the cases	3. Selection of controls	4. Definition of Controls	1. Comparability of Cases and Controls on the Basis of the Design or Analysis	1. Ascertainment of Exposure	2. Same method of ascertainment for cases and controls	3. Non-Response Rate
Yu *et al*.^37^			+				+	
Ho *et al*.^19^	+		+	++	+	+	+	
Huong *et al*.^38^	+	+		+		+	+	

Both “selection” and “exposure” were generally quite weak across the studies reviewed. Two studies used community control groups^19,37^ while one study had non-*S*. *suis* sepsis diagnosed patients as controls^38^. The definition of cases was explained sufficiently among studies with high and moderate score attainment^19,38^ but representativeness of the cases was stated only in one study^38^.

Only one study achieved a high “comparability” quality assessment score^19^. Neither of the two remaining studies adjusted for confounders^37,38^. Among these, one study used medical record for exposure ascertainment^38^ whereas the other used a questionnaire without blinding the interviewers to case and control status^37^. None of the studies provided the information concerning non-response rate or addressed on the issue.

The included randomized controlled study was low risk of bias based on RoB 2.0^10^. Support for the judgement was provided in supplementary appendix Table S2.

### Patient characteristics

Among 32 included studies, a total of 1,454 *Streptococcus suis* cases were reported. Majority of patients were men, comprising more than two-thirds of *S*. *suis* cases except in the study by Kerdsin *et al*. (2009) in which there was a relatively higher number of female patients compared to other studies^21^. Majority of cases were Asian particularly from Thailand, Vietnam and China whereas minority were patients from European countries where cases were largely occupation related. There were only 15 African patients derived from a population-based surveillance study in Togo^41^

*S*. *suis* patients were generally healthy adults before acquiring the infection. The mean age ranged between 37 to 63 years. A lower mean age was noted in 2 studies^16,28^. Mean age was reported in most studies^9,11,13–16,18,20,22–24,26,29–35,38^ whereas 6 studies reported the value in median^10,21,25,27,36,39^. Neither mean nor median age were reported in 3 studies^17,37,41^ (see supplementary appendix Table S1. Study key characteristics).

Study population were mainly *S*. *suis* meningitis identified from studies done in bacterial meningitis patients^9,10,13,15,19,22,23,28,29,35,41^ while the rest were patients diagnosed with *S*. *suis* infection^11,14,16–18,20,21,24–27,31–34,38–40,42^. Diagnosis was based on either standard bacterial culture or real-time polymerase chain reaction (RT-PCR) in most studies. However, *S*. *suis* probable or suspected cases defined as cases with compatible clinical illness without laboratory confirmation were also included in 3 studies^36,37,39^. Most human *S*. *suis* infections were caused by serotype 2 strain. An occurrence of serotype 14 infections were sporadically reported mainly from northern, Thailand^21^ whereas there were very few number of serotype 14 isolates identified in Vietnam^10^. Serotype 4 strain and untypeable serotype were considered to be rare^9^.

### Risk factors

Risk factors associated with acquiring *S*. *suis* infection included raw pork consumption, pig-related occupation, pigs or pork exposure, alcohol drinking, skin injury especially during pork exposure and underlying diseases contributing to immunocompromised conditions (supplementary appendix Table S1.). Although transmission by skin abrasion was believed to be the main route of infection, history of skin injury during exposure or before infection was noted only in some studies (9.5–100%) in which majority had a small percentage^9,11,14,23–25,31,32,36^.

Varying results were noted between studies concerning risk factors of the disease. Exposure to pigs or pork and related occupation were the major risk factors found in a number of studies^14,23,37,38,41^ whereas raw pork consumption or pig exposure was not present in around two-third of patients in other studies^10,16,28,31,33,35^. A high frequency of raw pork consumption was found among Thai patients especially in northern Thailand^26,39,40^. Although alcohol drinking was rarely reported in previous studies, a relatively high number of alcohol consumption was found in some studies from Thailand^26,29,40^.

Despite similar study design, the three case-control studies included in the meta-analysis demonstrated different features. The prospective case-control study conducted in Vietnam included patients with invasive *S*. *suis* infection as cases and two control groups; an unmatched hospital control group and a matched community control group by residency and age within a 10 years range, at a ratio of 1:3^19^ whereas the retrospective case-control study from the same country recruited *S*. *suis* infection patients as cases and non-*S*. *suis* sepsis diagnosed patients as controls^38^. A matched case-control study in Sichuan, China included *S*. *suis* infected patients in case group and individuals who had exposure with cases within 1 week prior to diagnosis as controls at a ratio of at least 1:1^37^. A standardized questionnaire was used to investigate on predisposing factors in two studies^19,38^. However, only in the prospective case-control study, the interviewers were blinded to case and control status^19^. In the matched case-control, the interviewers were not blinded and only 15 out of 29 patients were interviewed face-to-face whereas the rest were unconscious, and the questionnaires were responded by their relatives^37^. The medical records were used for the other study^38^.

Different case and control definitions were used among studies. A confirmed *S*. *suis* case was generally defined as an admitted patient with confirmed *S*. *suis* infection either by blood/CSF culture or real-time polymerase chain reaction (RT-PCR) in 2 studies^19,38^ whereas a case was defined as *S*. *suis* case confirmed by either laboratory or clinical diagnosis in one study^37^.

Community controls definition was quite similar in two case-control studies^19,37^ except they were randomly identified and matched by age in one study^19^ while one study only had hospital control group defined as confirmed non-*S*. *suis* sepsis patients during admission^38^.

### Meta-analysis

**Figure 2:**
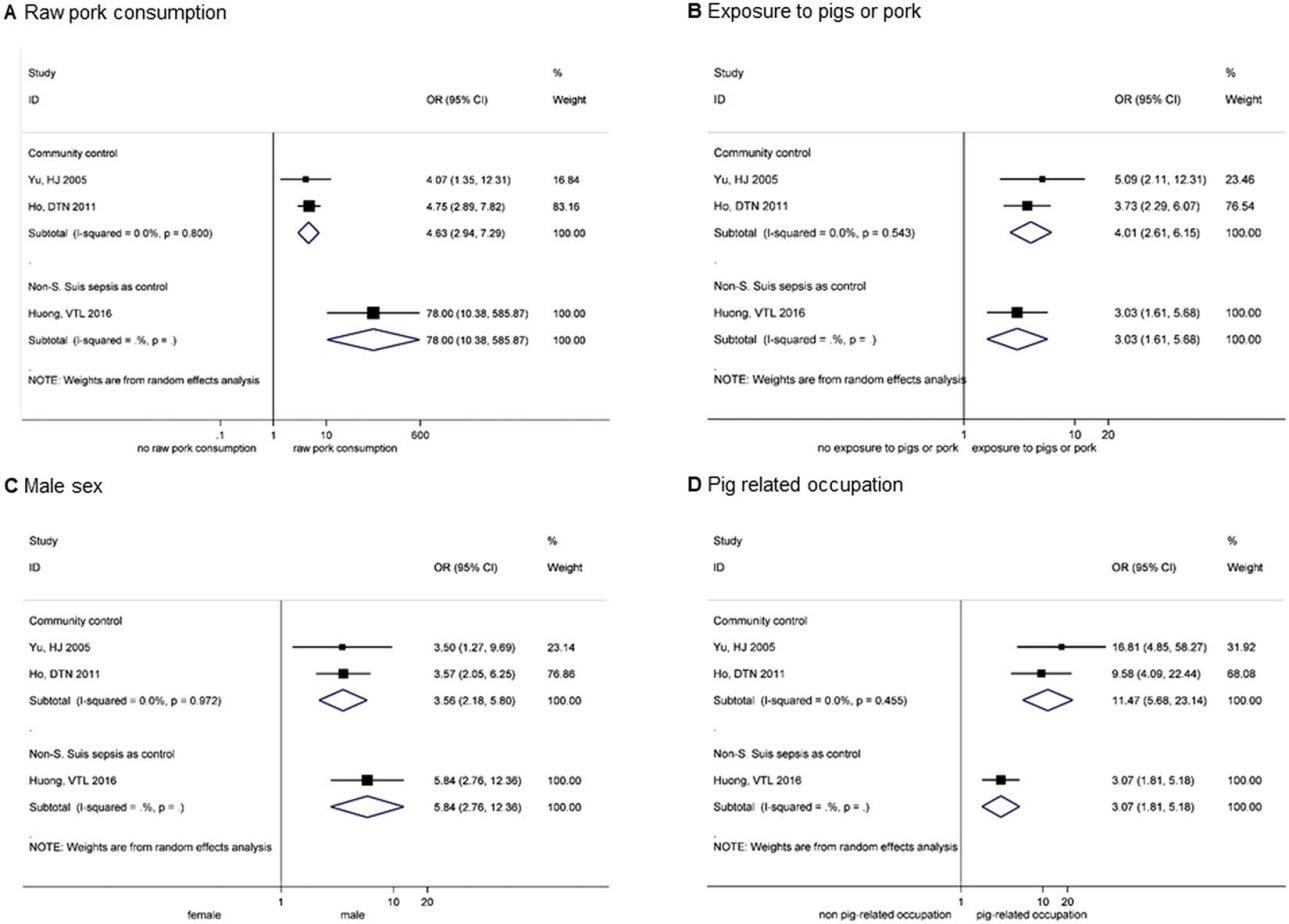
Risk factors of Streptococcus suis infection; Raw pork consumption (**A**), Exposure to pigs or pork (**B**), Male sex (**C**), and Pig related occupation (**D**). (**B**) *Note:* Individuals living in PRRS district or area adjacent to PRRS and those involved in pork cleaning, cutting and processing were used as proximate numbers for population exposed to pigs or pork in Huong *et al*. and Yu *et al*.^37,38^ respectively. (**D**) *Note:* In Yu *et al*., the number of those involved in slaughtering was used to represent pig-related occupation individuals^37^.

A total of 850 participants among 3 included case-control studies were analyzed by type of control groups (community controls and non-*S*. *suis* sepsis diagnosed cases). Major risk factors including raw pork consumption, exposure to pigs or pork, pig-related occupation and male sex were found to be significantly associated with *S*. *suis* infection according to all meta-analyses. Some proximate numbers were used due to different categorization of predisposing factors. The number of individuals living in Porcine Reproductive and Respiratory Syndrome (PRRS) district or adjacent area and those involved in pork cleaning, cutting and processing were used to represent population exposed to pigs or pork in the matched case-control and the retrospective case-control studies respectively^37,38^. For pig-related occupation, the number of those involved in slaughtering was used to represent pig-related occupation individuals under justification that most participants were farmers who were usually involved in slaughtering activity. The random-effects meta-analysis results on risk factors associated with *S*. *suis* infection were presented in Fig. 2A–D.

Raw pork consumption was significantly higher among cases than controls and much more pronounced in study by Houng *et al*. in which the control group was from non-*S*. *Suis* sepsis cases [pooled OR 4.63, 95% CI 2.94–7.29 and OR 78.00 95% CI 10.38–585.87, respectively] (Fig. 2A)^38^. Conversely, the overall estimate was stronger among studies with controls derived from community [pooled OR 11.47, 95% CI 5.68–23.14] for pig-related occupation whereas a weaker positive association was noted when the control group were non-*S*. *Suis* sepsis patients [OR 3.07 95% CI 1.81–5.18 (Fig. 2D). There was no significant heterogeneity observed between studies (I^2^ = 0.0%, p = 0.80 and I^2^ = 0.0%, p = 0.455 respectively).

The proportion of men was remarkably greater among cases in both analyses by type of control group [pooled OR 3.56, 95% CI 2.18–5.80 and OR 5.84 95% CI 2.76–12.36, respectively] (Fig. 2C)^19,37,38^ and was nearly 6 times higher in cases than controls in the study with controls drawn from hospital non-*S*. *suis* sepsis patients [OR 5.84 95% CI 2.76–12.36]^38^.

Pigs or pork exposure was around 3 to 4 times higher in cases than controls among studies with controls drawn from hospital non-*S*. *Suis* sepsis diagnosed patients and community respectively [OR 3.03, 95% CI 1.61–5.68 and pooled OR 4.01, 95% CI 2.61–6.15, respectively] (Fig. 2B)^19,37,38^. The results were consistent among all analyses and there was non-significant heterogeneity. It was seen that the studies with community controls^19,37^ generally showed more precise values compared with the study with controls drawn from non-*S*. *Suis* sepsis cases^38^.

### Clinical manifestations and outcomes

Meningitis was the most frequent clinical presentation, followed by septiceamia and arthritis in which an occurrence of cases subsequently developed sepsis arthritis were also reported (supplementary appendix Table S1). The spectrum of signs and symptoms of presentations were quiet similar across studies. Majority of meningitis patients developed classic meningitis symptoms including severe headache, high fever, neck stiffness and a change in mental status^9^. Petechiae or other skin abnormalities were present in few studies ranging between 3% to 7% among *S*. *suis* meningitis^9,10,22^.

Endocarditis was usually less common whereas endopthalmitis and spondylodiscitis were^38^ considered to be rare manifestations. In contrast, it was found that infective endocarditis was among or the most common clinical presentations found in two case series despite no underlying heart disease in most patients included^11,33^. The most frequent vegetation site found was aortic involvement^33^. The proportion of cases who developed toxic shock syndrome (TSS) was quite small approximately 2–28% in majority of studies^10,11,18,34–36,39^ except in 2 epidemiological studies in China (62% and 50%)^24,37^. Toxic shock syndrome (TSS) and subacute endocarditis (SBE) were found to be associated with high mortality rate according to a series of 43 patients from Thailand, 80% and 50% among patients with TSS and SBE respectively^18^. TSS cases were younger with shorter incubation period, a lower total serum protein and antibody levels compared to non-TSS patients^18^.

Incubation period was provided in 14 studies, the median time from exposure to onset ranged between 1 to 4.8 days^9–11,16–18,24,25,27,29,30,34,36,41^. Most infections occurred during the summer months^11,15,22,25,35,36^ or the rainy season^20,26,33^.

The disease mortality rate was low compared to meningitis caused by other agents (0–33.3%)^9–11,14,16–18,20–22,24–36,38,39,41^. However, deafness incidence was high in majority of studies and largely sequelae from meningitis syndrome (7–93%)^9–11,13–18,20–22,24–26,28–35,38,40,41^. Hearing loss was usually permanent once it already started even after successful meningitis treatment^22^. Vestibular dysfunction or ataxia was also common (8–80%)^11,17,24,26,29,32,35^ and present in half of meningitis cases in a case series^26^ whereas visual loss was noted in a few studies (4–60%)^17,35,41^.

Deaths were mainly resulted from other complications rather than meningitis including multiple organ failure^33^, disseminated intravascular coagulation (DIC)^22,31^, bacterial peritonitis, sepsis and infective endocarditis^31^. Relapse rate was small and normally successfully treated with continuation of penicillin or combination therapy^9^.

### Treatments

Most *S*. *suis* isolates were sensitive to penicillin or cephalosporins^10,11,14,15,22,25,29,33,41^. Treatment with high dose intravenous Penicillin G were highly effective in majority of patients^22^. The mean of the minimum inhibitory concentration (MIC) for penicillin ranged from 0.015 to 0.06 mg/mL^11,14,15,22,31,33^. Tetracycline and macrolide resistance was common^10,11,14,25,41^ whereas few cases with multiple antimicrobial resistance was noted^31^.

Mean treatment duration ranged from 7 to 42 days^10,11,13,16,20,21,27,29–31,34–36,38^. Longer treatment duration was usually needed in case of complications including meningitis, spondylocitis and endocarditis^31,33^. Combination regimen including penicillin or cephalosporin plus aminoglycoside was found to be effective in treating infective endocarditis^33^.

Adjuvant therapy with dexamethasone was found to reduce the risk of hearing loss and neurological complications according to the randomized double-blind, placebo-controlled trial included^10^. In contrast, the effect of steroid against hearing loss protection could not be established according to the two included case series^26,35^.

## Discussion

*S*. *suis* infection were predominantly found in adult male patients and the elderly^22^. The absence of pediatric infections was probably due to a lack of exposure to associated risk factors among children. A relative high proportion of male cases can be explained by the fact that the disease is occupation related. The more likelihood of exposure to pigs and predisposing factors such as raw pork consumption, slaughtering activity and alcohol use has posed men to be more prone to infection through their risk behaviors. However, more female patients were also identified despite no history of pigs or pork exposure^21^. According to the meta-analysis by type of control group of the included case control studies, pig-related occupation, exposure to pig or pork, male sex and raw pork consumption are significantly associated with *S*. *suis* infection^19,37,38^. However, a definite history of contact with pigs or pork could be elicited only in few number of patients^22^. Unknown skin lesions may contribute to the entry of organism^9,22^. The nature of recalled bias or missing data in retrospective studies included was also partly responsible for this finding. Indirect exposure was as well possible due to widely availability of wet markets in Asian countries. The possibility of oral route transmission has been raised which might explain the diarrhea found in some cases^11^. It was seen that raw pork consumption was significantly associated with *S*. *Suis* infection and the proportion of cases with raw pork consumption was substantially higher than controls drawn from non-*S*. *Suis* sepsis patients in study by Houng *et al*.^38^. The notably higher number was potentially subject to information bias as doctors would have more likely asked patients with *S*. *suis* infection concerning this particular exposure compared to others. Cultural food habits involving consumption of raw pork and pig’s blood with alcohol drink could be a reason of the high frequency of infection among Thai population^18,26^. According to a study on impact of a food safety campaign in the Phayao Province in northern Thailand, the disease incidence significantly declined after the first two years upon campaign implementation. However, the infection rate rose again in the third year which implied the existence of deep-rooted cultural behavior of raw pork consumption and the need of an effective public health education program to eliminate the risk of infection^42^.

Other key characteristics that make patients vulnerable to infection include splenectomy^10,19,32^, alcohol drinking or alcoholic liver disease^9,11,18–20,25–29,31–35,38,40^, concurrent diabetes^11,18,19,25–28,33,34^, renal or pulmonary tuberculosis^11,25^, cancer^9,25,29,33^, heart disease^16,18,27,29,31,33^, on corticosteroid^29^ which contribute to immunocompromised condition. There were around 50% and 80% of *S*. *suis* patients with underlying diseases found in two case series^18,33^. A high disease burden of rheumatic heart disease in Asia^43^ combined with pig exposure which is an established risk factor may contribute to the high rate of infection in this region. Although there was no *S*. *suis* infection in pregnancy reported in literatures, according to the included retrospective cohort study, one of the dead cases was a 40-year old pregnant woman with 2-month-gestational age who presented with septic shock, DIC and died within 24 hours of admission^34^. This suggests that pregnancy may cause a patient to be in a more susceptible condition resulting in fatal outcome.

Meningitis is the most common clinical characteristic found in *S*. *suis* infection and is usually accompanied with hearing loss. This concomitant morbidity warrants the need of close monitoring and early adequate care in meningitis patients^34^. However, it should be noted that most studies included were done in meningitis population. The invasive nature of *S*. *suis* serotype 2 which is the major serotype found in human infections may be relevant. The polysaccharide capsule containing sialic acid feature makes the organism become highly invasive in entering to blood stream and penetration to blood-brain barrier^44^.

Deafness appears to be the most common sequelae found among survivors. The mechanism of hearing loss is probable from *S*. *suis* invasion to the perilymph via cochlear aqueduct resulting in suppurative labyrinthitis according to animal experiments^45^. The benefit of dexamethasone treatment in hearing loss prevention was demonstrated in the included randomized control trial study^10^. However, inconsistent findings were shown in the two case series^26,35^. This was probably due to a small number of patients and varying degree of deafness severity^26,35^. Notwithstanding this contradiction, it should be justified to use corticosteroid as an adjunctive therapy based on the high rate of hearing impairment in *S*. *suis* meningitis.

The high frequency of STSS and fatality rate was found in the China outbreak^36^, but, by contrast a lower proportion of STSS and mortality was reported in the outbreak investigation in Thailand^39^. The marked contrast in clinical outcome and severity could be due to the different main risk factors. In the Sichuan outbreak, pig slaughtering and widespread of porcine disease were the leading causes whereas consumption of raw pork was the major risk factor in the Thailand outbreak^36,39^. STSS was a significant risk factor of mortality with a rapid onset^18,34^. There were significant shorter incubation period and hospital stay among patients with STSS in which majority died within 24–72 hours upon admission^18^.

The disease mortality was low. The number of *S*. *suis* meningitis case-fatality rate was lower compared to other meningitis among the same age group population^5,46,47^. This might be due to most *S*. *suis* infected individuals were healthy adults with less frequent predisposing conditions whereas other bacterial meningitis patients usually presented with underlying diseases^47^ which have been correlated with a poor prognosis^9^.

The number of *S*. *suis* cases appears to be higher during the summer or rainy seasons. The high occurrence of infection in the hot and humid weather is believed to be a precipitating factor that triggers more stress on pigs during transportation^15^. Apart from that the condition also enable the organism to proliferate in pig carcasses increasing infectivity during contaminated meat exposure^15^.

To the best of our knowledge, this is the first systematic review and meta-analysis with the primary aim to comprehensively explore on the risk factors acquiring *Streptococcus suis* infection in human whereas the previous systematic review^48^ focused only among studies on *S*. *Suis* meningitis. We searched extensively in 8 major databases and included all studies with at least 4 *S*. *Suis* infection cases with no time nor language restrictions while the previous systematic review included only studies written in West-European languages published between January 1, 1980 and August 1, 2015 and described at least 5 adult *S*. *suis* meningitis patients in whom at least one described clinical characteristic^48^. A rigorous quality assessment was done for both included observational and randomized controlled studies as well as comprehensive critical appraisals on all 32 studies included.

Some limitations of this review can be noted. The studies included in the review were largely descriptive and very diverse in terms of study designs and quality resulting in a small number of studies could be utilized for meta-analysis. The variability in the methodology and quality of the three case-control studies particularly the different types of control group caused some challenges whether they were combinable. However, we took consideration of this potential clinical heterogeneity and performed meta-analysis by control group. There was no significant heterogeneity seen in our analyses and the odd ratios were towards the same direction in favoring increasing risk of *S*. *suis* infection in cases than controls. The studies with community controls were generally showed more precise results^19,37^ compared with values from the study with controls obtained from hospital^38^ which might be due to larger sample size and selection of controls. Given the report with at least 4 cases of the inclusion criteria, there may be underreported cases. However, with the fact that the data is highly heterogeneous especially in different population and the primary outcome of interest is risk factors associated with the infection. All the main risk factors should have been identified based on the study inclusion criteria. In addition, majority of articles reviewed were retrospective studies which could have been potentially to recall bias and missing data. Finally, some proximate numbers were used in meta-analysis on risk factors. However, these could be the closest estimated numbers to be drawn according to the defined definition based on the authors’ judgement under this limitation.

## Conclusion

*S*. *suis* infection is not uncommon. The low number of cases reported were largely due to under diagnosis and unawareness of the disease. The organism is often misidentified by clinicians resulting in delay or inadequate treatment. It is important that patients with suggestive *S*. *suis* clinical symptoms with predisposing risk factors should receive adequate care while waiting for laboratory confirmation despite negative bacterial culture either due to misidentification or previous antibiotic administration. Developing a screening protocol would be useful to aid the treatment decision. Once a clear clinical picture is identified, the diagnosis should not be too difficult. The immediate treatment with penicillin or antimicrobial that the pathogen is susceptible to before development of complications particularly deafness would be essential in preventing long term mortality and morbidity.

In an absence of vaccination, the best control measure is to prevent the disease transmission. Public health interventions including a food safety campaign would be effective to enhance understanding about the disease especially in settings where there is a strong relationship between raw pork consumption and traditional culture.

## Methods

The study reporting methodology was done according to the Preferred Reporting Items for Systematic Reviews and Meta-analyses (PRISMA) statement^49^. The study protocol has been registered in PROSPERO under protocol number CRD42018083596 (https://www.crd.york.ac.uk/prospero/display_record.php?RecordID=83596).

### Search strategy and study selection

A number of relevant electronic databases were systematically searched including CINAHL plus, Cochrane, EMBASE, Global Health, Grey literature, Ovid Medline, PubMed, and Science Direct. The MeSH terms used were “Streptococcus suis” OR “Streptococcus suis AND “infection” limited in human with no time nor language restriction.

The primary outcomes were risk factors associated with *Streptococcus suis* infection. The secondary outcomes were clinical presentations and outcomes of the disease. Articles were included if there were risk factors with or without clinical characteristics or outcomes of *Streptococcus suis* infection in human in which the cause of the disease was explained and at least 4 patients was described. Review, systematic review and meta-analysis articles as well as publications reporting overlapping data with the included articles were excluded. The references cited in the identified articles were also reviewed and judged to be included in case they deemed relevant. The final searched was done on September 18, 2017.

### Data Extraction

The inclusion criteria were confirmed by AR and TMK. The data was searched, screened and extracted by one reviewer (AR) and confirmed by either BHG, LHL, TMK or SS for articles in English and Thai. For studies in other languages, BHG extracted the data from articles in Chinese with confirmation by LHL. The data from the article in Croatian language was extracted by TMK and confirmed by a native speaker together with the English abstract. The search strings used can be referred to Appendix 1. The consultation process was employed in case of doubts or disagreements to reach a consensus between reviewers and all authors.

For articles containing ambiguous data, two email attempts to the corresponding authors were carried out for clarification. The studies were excluded for analyses if there was no response received. In case the study had primary data published elsewhere, the previous publications were also checked and verified. Alternatively, an attempt to obtain clarification from the first or corresponding author would be made.

### Risk of bias assessment

The quality of nonrandomized studies included were assessed according to the Newcastle-Ottawa Scale (NOS)^50^. The randomized controlled study^10,51^ was assessed using a revised tool to assess risk of bias in randomized trial (RoB 2.0) which was based on the Cochrane Collaboration Approach^52^. The main study previously published^51^ was also referred to where there was no information mentioned in the included article^10^.

Information including predisposing factors, patient demographics, clinical manifestations, treatment and outcomes were extracted.

### Meta-analysis

Meta-analysis for risk factors was carried out for case-control studies using STATA 14.2 (College Station, Texas, USA). The analyses by type of control groups (community control, and non-*S*. *suis* sepsis) were done as this variation could potentially be the source of clinical heterogeneity. Results for the association between risk behaviors and *streptococcus suis* infection were pooled using random-effects model in order to account for heterogeneity^53,54^.

Forest plots were used to display the effect sizes (ES) from each study with their relevant 95% confidence intervals’ and overall estimated ES. Heterogeneity was tested using I^2^ and Q statistics^53,54^.

Four main predisposing factors were defined in analyses: (1) Exposure to pigs or pork, defined as history or recalled of exposure with pigs or pork before illness without slaughtering, (2) Pig-related occupation includes farmer, butcher, abattoir worker, seller of raw pork, (3) Consumption of raw pork, defined as consumption of raw or partially cooked pork including swine materials, and (4) Male sex. In case the number of the defined category was not provided, the relevant number which could be assumed to be similar or in closest category would be utilized. Community controls were selected as control group in order to derive the same population who actually would have been cases if the outcome was present except in the retrospective case-control study which was designed to have only hospital control group^38^.

## Electronic supplementary material

Supplementary information
