# Supplementary material for: Risk factors for Streptococcus suis infection: A systematic review and meta-analysis
Source: Sci Rep. 2018 Sep 6;8:13358. doi: 10.1038/s41598-018-31598-w (PMC6127304; doi:10.1038/s41598-018-31598-w)
Supplement: Supplementary file 1 — Supplementary information [file 41598_2018_31598_MOESM1_ESM.docx]

# **Supplementary appendix**

# **Risk factors for *Streptococcus suis* infection: A systematic review and meta-analysis**

Ajaree Rayanakorn^1^, Bey-Hing Goh^1-2^, Learn-Han Lee^1-2^, Tahir Mehmood Khan^1,4^, Surasak Saokaew^1-3^

1 School of Pharmacy, Monash University Malaysia, Bandar Sunway, Selangor Darul Ehsan, Malaysia

2 Center of Health Outcomes Research and Therapeutic Safety (COHORTS), School of Pharmaceutical Sciences, University of Phayao, Phayao, Thailand

3 Center of Pharmaceutical Outcomes Research (CPOR), Faculty of Pharmaceutical Sciences, Naresuan University, Phitsanulok, Thailand

4 The Institute of Pharmaceutical Sciences (IPS), University of Veterinary & Animal Sciences (UVAS), Outfall road, Lahore, Pakistan

**Supplementary Content**

[Appendix 1 The search strings used 3](#_Toc461527261)

[Table S1. Study key characteristics. 4](#_Toc461527262)

[Table S2. Risk of bias assessment (RoB2.0) detailed notes 16](#_Toc461527262)

**Appendix 1: The search strings used**

**EMBASE (Ovid)**

streptococcus suis.mp. [mp=ti, ot, ab, sh, hw, kw, tx, ct]

limit 1 to humans

("streptococcus suis" and streptococcus suis and infection).mp. [mp=ti, ot, ab, sh, hw, kw, tx, ct]

limit 3 to humans

**PubMed**

("streptococcus suis"[MeSH Terms] OR ("streptococcus"[All Fields] AND "suis"[All Fields]) OR "streptococcus suis"[All Fields]) OR (("streptococcus suis"[MeSH Terms] OR ("streptococcus"[All Fields] AND "suis"[All Fields]) OR "streptococcus suis"[All Fields]) AND ("infection"[MeSH Terms] OR "infection"[All Fields])) AND "humans"[MeSH Terms]

**CINAHL plus (via EBSCO)**

Streptococcus suis OR ( Streptococcus suis AND infection ) AND humans

**Medline (Ovid)**

(streptococcus suis or streptococcus suis and infection).mp. [mp=title, abstract, original title, name of substance word, subject heading word, keyword heading word, protocol supplementary concept word, rare disease supplementary concept word, unique identifier, synonyms]

limit 1 to humans

**Science Direct**

(streptococcus suis) or (streptococcus suis and infection), limit to humans

**Cochrane (via Ovid)**(("streptococcus suis" or streptococcus suis) and infection).mp. [mp=ti, ot, ab, sh, hw, kw, tx, ct]

limit 1 to humans

**Global Health**

((streptococcus suis) OR (Streptococcus suis and infection) AND (humans))

**Grey literature (Greynet)**

Streptococcus suis OR "Streptococcus suis AND infection"

| Study | Country | Study design | Sample size | Mean age (yr) (range) | Male (%) | Major risk factors % | | | | Main clinical presentations (%) | Treatment | Outcomes  (%) |
| --- | --- | --- | --- | --- | --- | --- | --- | --- | --- | --- | --- | --- |
|  |  |  |  |  |  | Recent exposure with pigs/porkª | Related occupation^b^ | Raw pork consumption^c^ | Others |  |  |  |
| Mai et al. 2008 ^10^ | Vietnam | A randomized, double-blind, placebo-controlled trial | 151  *Note:* 76 received dexamethaxone, 72 received placeo, 3 received neither All patients initially received Cetriaxone 2 g q 12 h | 46.5* (19-84) | 77.5 | 33.1 | 8.6 | NR | 2 splenectomy | 100 Meningitis¶ | 100 ceftriaxone 2 g q 12 h  50.33 Dexamethasone | 2.6 Death  Complete recovery: Dexa gr 36.8% vs. placebo gr. 38.7%  66.4 Hearing loss: 34.4 temporary, 41 permanent |
| Khin Thi & Chan 1985 ^22^ | Hong Kong | Case series | 30 | 58.7 (23-84) | 66.7 | 23.3 | NR | NR | NR | 86.67 Meningitis  26.67 Diarrhea  16.67 Arthristis  13.33 Sepsis  6.67 DIC & petechiae  3.33 Endopthalmitis | Penicillin G | 23.3 Death  50 Hearing loss |
| Kay, R et. al. 1995 ^45^ | Hong Kong | Case reports & case series | 25 | 55 (20-75) | 64 | NR | 60 | NR | 16 Skin injury up to 16 dys before admission  12 Alcohol drinking  8 Concurrent DM  4 Renal tuberculosis | 84 Meningitis  24 Arthritis  4 Sepsis  4 SBE  4 Septic shock | IV Penicillin G  IV Penicillin G+gentamicin | 4 Death  64 Hearing loss  40 Vestibular dysfunction  44 Vertigo/ ataxia |

**Table S1. Study key characteristics**

| Study | Country | Study design | Sample size | Mean age (yr) (range) | Male (%) | Major risk factors % | | | | Main clinical presentations (%) | Treatment | Outcomes  (%) |
| --- | --- | --- | --- | --- | --- | --- | --- | --- | --- | --- | --- | --- |
|  |  |  |  |  |  | Recent exposure with pigs/porkª | Related occupation^b^ | Raw pork consumption^c^ | Others |  |  |  |
| Dragojlovic et. al. 2005 ^17^ | Serbia | Case series | 5 | NR (22-63) | 100 | 100 | NR | NR | NR | 100 Meningitis  20 Sepsis  20 SBE  20 Endothalmitis  20 Respiratory failure  20  Thrombocyto  penia | 2^nd^ and 3^rd^ generation Cephalosporins (80%)  Aminoglycosides (20%) | No death  20 Complete recovery  80 Recovery with permanent hearing loss |
| Yu, HJ et al. 2005 ^37^ | China | A matched case-control study | 29/147 (cases/ controls) | NR (36-72) | 82.8/ 57.8 (cases/ controls) | 72.4/34 (21/50)    *Note:* Calculated from individuals involved in cleaning, cutting, processing of raw pork | 89.66/34 (26/50)  *Note:* Calculated from individuals involved in pig slaughtering | 86.2/60.5 (25/89) | NR | 27.6 Meningitis  10.34  Sepsis  62 STSS | NR | NR |
| Yu, H et al. 2006 ^36^ | China | Outbreak investigation report | 215** | 54* (26-82) | 84 | 28 | 96  *Note:* All were farmers. | 0 | 65 Sick pig/goat slaughtering  48 Skin injury during exposure | 48 Meningitis  28 STSS  24 Sepsis | NR | 18.14 Death |

| Study | Country | Study design | Sample size | Mean age (yr) (range) | Male (%) | Major risk factors % | | | | Main clinical presentations (%) | Treatment | Outcomes  (%) |
| --- | --- | --- | --- | --- | --- | --- | --- | --- | --- | --- | --- | --- |
|  |  |  |  |  |  | Recent exposure with pigs/porkª | Related occupation^b^ | Raw pork consumption^c^ | Others |  |  |  |
| Fongcum et. al. 2009 ^18^ | Thailand | Case series | 43  *Note*:  10 patients from retrospective review during the outbreak were excluded from clinical presentation and outcomes | 50 | 92.5 | 7 | NR | 88.7 | 83 Underlying diseases | 37.21 Meningitis  27.91 Sepsis  23.26 STSS  9.3 SBE  2.33 Spondylodiscitis | NR | 27.9 Death  46.5 Complete recovery  18.6 Permanent deafness  7 Disability |
| Wertheim et. al. 2009 ^35^ | Vietnam | Case series | 50 | 48 (17-78) | 88 | 10 | 70 | 6 | 26 Alcohol drinking | 100 fever  88 Neck stiffness  84 Kernig’s sign  92 headache  46 confusion  16 Respiratory failure  14 Skin rash  12 STSS | Ceftriaxone in combination with ampicillin  52% received corticosteroid | 6 Death  52 Complete recovery  42 Recovery with sequelae  38 Hearing loss  4 Paralysis  4 Loss of vision  4 Dysarthria with gait ataxia |

| Study | Country | Study design | Sample size | Mean age (yr) (range) | Male (%) | Major risk factors % | | | | Main clinical presentations (%) | Treatment | Outcomes  (%) |
| --- | --- | --- | --- | --- | --- | --- | --- | --- | --- | --- | --- | --- |
|  |  |  |  |  |  | Recent exposure with pigs/porkª | Related occupation^b^ | Raw pork consumption^c^ | Others |  |  |  |
| Kong et. al. 2009 ^23^ | China | Epidemiological analysis | 4 | 47.3 | 100 | 100 | 50 | NR | 100 Skin injury | 100 Meningitis¶ | NR | 100 Fever  100 Headache  100 Meningeal irritation  50 Vomiting  25 each Coma, cramping and chilling |
| Ho et. al. 2011 ^19^ | Vietnam | Prospective case-control study | 101/303/ 300 (cases/ hospital controls/ community controls) | 50 (41-59)/ 27 (20-40)/ 50 (41-60) | 82.2/ 66.7/ 56.3 | 45.5/12.9/18.3  (46/39/55) | 20.8/2.6/2.7 (21/8/8) | 47.5/21.8/16 (48/66/48) | 32.7/5.9/3.7 Skin injury  13.9/5.9/6.7 Alcoholism  3/1/1.3 DM  1/0/0 Splenectomy | NR | NR | NR |
| Huong et. al. 2016 ^38^ | Vietnam | Retrospective case-control study | 90/183 (cases/ controls)  *Note:* Cases: *S.Suis* case patients  Controls: non-*S.suis* sepsis controls | 48.5 (46.2-50.8)/ 50.6 (48.2-53.1) | 90/ 60.7 | 83.3/62.3 (75/114)  *Note:* Derived from those living in PRRS district or area adjacent to PRRS^ district | 64.4/37.2 (58/68) | 30/0.5 (27/1) | 31.1/21.3 Alcoholism | 86.7/0 Neck stiffness  0/3.3 SBE, p=0.09  5.7/29.5 STSS, p=0.001  22.2/19. Skin rash  16.7/0.5 Purpura fulminans, p<0.001  15.9/28.4 ARF, p = 0.025  5.7/8.7 ALF  5.7/12.6 ARDS, p=0.08  4.5/2.7 Coagulopathy | Corticosteroid received 75%/11.5%, p= 0.001 | 6.6/25.6 Death  28.9/4.4 Recovery with sequelae  16.7/0 Hearing loss  8.9/0 Tinnitus |
| Study | **Country** | **Study design** | **Sample size** | **Mean age (yr) (range)** | **Male (%)** | **Major risk factors %** | | | | **Main clinical presentations (%)** | **Treatment** | **Outcomes**  **(%)** |
|  |  |  |  |  |  | Recent exposure with pigs/porkª | Related occupation^b^ | Raw pork consumption^c^ | Others |  |  |  |
| Tall et al. 2016 ^41^ | Togo | A public health surveillance study | 15 | NR (5-≥50) | 80 | 80 | 66.7 | 93.3  *Note*: Pork consumption, 9 patients at least once per week | 20 Pig famers involving slaughtering | 100 Meningitis¶ | NR | 7 Death  13.33 Complete recovery  80 Recovery with sequelae  67 Hearing loss  42 Visual impairment  17 Paralysis |
| Takeuchi et. al. 2017 ^42^ | Thailand | A descriptive study on food safety campaign | 71  *Note:* Before the campaign: 31  After the campaign: 41 | 56.7 | 71.8 | 18.3 | NR | 78.9 | NR | 53.5 Meningitis | NR | 9.9 Death  29.6 Hearing loss |
| Arends et. al. 1988 ^9^ | The Netherlands | Case series | 30^^ | 49 (21-76 | 86.7 | 6.7 | 83 | NR | 63.3 Skin injury  6.7 Skull fracture/ operation  6.7 Carcinoma  3.3 Alcohol abuse  3.3 Zollinger-Ellison syndrome  3.3 Cerebral confusion | 100 Meningitis¶  6.7 Shock  6.7 Petechiae  3.3 Macular bleeding/ arthritis | Ampicillin/ Penicillin | 6.7 Death  6.7 Relapse  *Note:* Penicillin MIC: 0.04 mg/mL and successfully treated with penicillin continuation  54 Hearing loss |

| Study | Country | Study design | Sample size | Mean age (yr) (range) | Male (%) | Major risk factors % | | | | Main clinical presentations (%) | Treatment | Outcomes  (%) |
| --- | --- | --- | --- | --- | --- | --- | --- | --- | --- | --- | --- | --- |
|  |  |  |  |  |  | Recent exposure with pigs/porkª | Related occupation^b^ | Raw pork consumption^c^ | Others |  |  |  |
| Walsh et. al. 1992 ^32^ | The UK | Case series | 35 | 47.3 (19-87) | 97.1 | NR | 82.9 | NR | 14 Skin injury  2.9 Drinking alcohol  2.9 Splenectomy | 85 Meningitis classic triad¶¶  60 Cellulitis  53 Arthritis  5.7 Meningitis  2.9 Endophthalmitis | Penicillin G, Gentamicin, Choramphenical | 13 Death  57.1 Recovered with sequelae  50 Hearing loss  30 Vertigo and ataxia  2.9 Relapse |
| Donsakul et. al. 2003 ^16^ | Thailand | A retrospective review | 8 | 39.5 (19-75) | 87.5 | 25 | NR | NR | 25 Ventricular septal defect | 76 Meningitis  25 Endocarditis  12.5 Arthritis | IV Penicillin (7/9) 18-24mU/dy, 2-6 wks or Ampicillin (1/8) 12 g/dy, 4 wks | 37.5 Complete recovery  62.5 Recovery with sequelae  62.5 Hearing loss |
| Suankratay et. al. 2004 ^29^ | Thailand | Case reports and case series | 12 | 49.5 (27-75) | 75 | 41.7 | 25 | NR | 75 Alcohol drinking  16.7 On corticosteriod  33.3 Underlying disease:  - 8.3 Urinary bladder cancer  - 8.3 RHD  - 8.3 Cerebrovascular accident  - 8.3 Chronic arterial insufficiency | 83.3 Meningitis  66.7 Meningitis classic triad¶¶  66.7 Skin and soft tissue infection  41.7 Severe myalgia  16.7 Shock  16.7 Sepsis  16.7 Arthritis | IV Penicillin G  IV Cefotaxime | 8.3 Death  58.3 Hearing loss  8.3 Vestibular dysfunction  8.3 Cerebritis/ ventriculitis  8.3 Relapse |

| Study | Country | Study design | Sample size | Mean age (yr) (range) | Male (%) | Major risk factors % | | | | Main clinical presentations (%) | Treatment | Outcomes  (%) |
| --- | --- | --- | --- | --- | --- | --- | --- | --- | --- | --- | --- | --- |
|  |  |  |  |  |  | Recent exposure with pigs/porkª | Related occupation^b^ | Raw pork consumption^c^ | Others |  |  |  |
| Chang et. al. 2006 ^14^ | Japan | Case series | 7 | 53.7 (47-58) | 57.1 | 100 | NR | NR | 71.4 Skin injury | 71.4 Meningitis  57.1 DIC  42.9 Sepsis  42.9 Arthritis  28.6 Endophthalmitis  14.3 Epidural abscess | NR  *Note*: All *S. suis* isolates were susceptible to penicillin, cefotaxime, ciprofloxacin | 14.3 Death  71.4 Deafness |
| Wangkaew et. al. 2006 ^33^ | Thailand | Case series | 41 | 51 (27-77) | 78 | 7.3  *Note:* Hx of raw beef consumption | 4.9 | 24.4 | 34.1 Alcohol drinking  7.3 Heart diseases  4.9 Concurrent DM  2.4 Stomach cancer | 39 Infective endocarditis 31.7 Meningitis  24.4 Sepsis  2.4 Spondylodiscitis  2.4 Endophthalmitis | Penicillin/ Cephalosporin+Aminoglycoside  *Note:* In infective endocarditis patients  IV Cefotaxime  Ceftriaxone  IV Penicillin G | 19.5 Death  30 Hearing loss |
| Lin et. al. 2007 ^24^ | China | Case series | 6 | 54.5 | 100 | NR | 100 | NR | 83.3 Skin injury  33.3 Slaughtering | 50 Meningitis  50 STSS | IV Penicillin G | 33.3 Death  33.3 Complete recovery  33.3 Recovered with sequelae: - 16.7 slow response - 16.7 Hearing loss and vestibular dysfunction |
| Study | **Country** | **Study design** | **Sample size** | **Mean age (yr) (range)** | **Male (%)** | **Major risk factors %** | | | | **Main clinical presentations (%)** | **Treatment** | **Outcomes**  **(%)** |
|  |  |  |  |  |  | Recent exposure with pigs/porkª | Related occupation^b^ | Raw pork consumption^c^ | Others |  |  |  |
| Beek et. al. 2008 ^13^ | The Netherlands | Case reports | 4 | 51 (33-64) | 75 | NR | 100 | NR | NR | 100 Meningitis¶  25 Seizure  25 Focal neurologic deficits | Amoxicillin or penicillin 14 days (3/4)  Ceftriaxone and stepped down to penicillin 14 days (1/4) | 25 Complete recovery  75 Permanent hearing loss |
| Ma et. al. 2008 ^25^ | Hong Kong | Case series | 21 | 61.1 (26-89)  *Note:* Median:62 years | 86 | 43 | 24 | NR | 9.5 Skin injury  29 Underlying disease:  - 14.3 DM  - 4.8 Alcoholism  - 4.8 Pulmonary tuberculosis  - 4.8 Breast carcinoma | 48 Meningitis  38 Sepsis  14 Endocarditis  9.5 Arthritis | Penicillin | 5 Death  19 Hearing loss |
| Rusmeechan et. al. 2008 ^28^ | Thailand | Case series | 41 | 37 (21-80) | 68.3 | NR | NR | NR | 20 Alcoholism  12 DM | 100 Meningitis¶  95 Meningitis classic triad¶¶  54 Abnormal mental status  2.4 Hemiparesis  29 Ataxia | Penicillin or 3^rd^ generation cephalosporins | 93 Hearing loss  No death |

| Study | Country | Study design | Sample size | Mean age (yr) (range) | Male (%) | Major risk factors % | | | | Main clinical presentations (%) | Treatment | Outcomes  (%) |
| --- | --- | --- | --- | --- | --- | --- | --- | --- | --- | --- | --- | --- |
|  |  |  |  |  |  | Recent exposure with pigs/porkª | Related occupation^b^ | Raw pork consumption^c^ | Others |  |  |  |
| Wangsom boonsiri et.al.2008 ^34^ | Thailand | A descriptive study (Retrospective cohort study) | 66 | 52.9 | 68.2 | NR | 6 | 59 | 11 Alcoholic liver disease  6 DM | 52 Meningitis  27 Sepsis  12 STSS  8 Endocarditis  1.5 Septic arthritis | Penicillin or ceftriaxone | 17 Death  29 Hearing loss  6 DIC  5 CHF  3 Intracerebral hemorrhage  1.5 Endophthalmitis  1.5 Subdural empyema  1.5 Peritonitis  1.5 Intervertebral discitis |
| Kerdsin et.al. 2009 ^21^ | Thailand | A retrospective review | 12 | 62.9* (40-79) | 58.3 | NR | 0 | 16.7 | NR | 58.3 Meningitis  25 Septic arthritis  16.7 Sepsis | NR | 41.7 Hearing loss  8.3 Acute respiratory distress syndrome  8.3 Extradural and subdural abscess  No death |

| Study | Country | Study design | Sample size | Mean age (yr) (range) | Male (%) | Major risk factors % | | | | Main clinical presentations (%) | Treatment | Outcomes  (%) |
| --- | --- | --- | --- | --- | --- | --- | --- | --- | --- | --- | --- | --- |
|  |  |  |  |  |  | Recent exposure with pigs/porkª | Related occupation^b^ | Raw pork consumption^c^ | Others |  |  |  |
| Navacharoen et. al. 2009 ^26^ | Thailand | Case series | 40 | 55 (27-82) | 75 | 25 | 52 | 62.5 | 49 Alcohol drinking  25 DM  5 Cirrhosis  5 HT  2.5 hyperlipidaemia | 47.5 Meningitis  27.5 Sepsis  25 Endocarditis | NR | 20 Death  27.5 Hearing loss:  - 15 Permanent hearing loss  50 Vestibular dysfunction  2.5 Visual impairment |
| Kerdsin et. al. 2011 ^20^ | Thailand | A retrospective review | 158 | 56.6 (18-98) | 72.8 | 7 | NR | 32.9 | 21 Alcohol abuse | 58.9 Meningitis  35.4 Sepsis  3.2 Septic arthritis  1.9 Infective endocarditis  0.6 Bacterial pneumonia  17.1 Diarrhea  22.2 Altered consciousness  5.7 STSS | IV Antibiotics e.g. ceftriaxone  Corticosteroid received 2.5% | 9.5 Death  21.5 Hearing loss |

| Study | Country | Study design | Sample size | Mean age (yr) (range) | Male (%) | Major risk factors % | | | | Main clinical presentations (%) | Treatment | Outcomes  (%) |
| --- | --- | --- | --- | --- | --- | --- | --- | --- | --- | --- | --- | --- |
|  |  |  |  |  |  | Recent exposure with pigs/porkª | Related occupation^b^ | Raw pork consumption^c^ | Others |  |  |  |
| Praphasiri et. al. 2015^27^ | Thailand | A public health surveillance study | 38 | 50* (23-73) | 73.7 | 26.3 | 33 | 65.8  *Note:* Included 13 patients involved in slaughtering who ate pork but could not recall how the meat was processed. | 53 Drinking alcohol  12 Underlying disease:  - 10.5 HT  - 7.9 DM  - 7.9 Alcoholism  - 2.6 Heart disease  - 2.6 Gout | 63.2 Meningitis  26.3 Sepsis  11 Arthritis | IV Cefotaxime | 31.6 Permanent deafness  68 Complete recovery  No death |
| Vilaichone et. al. 2002^31^ | Thailand | Case series | 17 | 46.2 (1 mth-75 yrs) | 64.7 | 17.7 | 2 | 5.8 | 11.8 Skin injury  17.7 Alcoholics  5.9 Congenital hydrocephalus  23.5 RHD | 52.9 Meningitis  11.8 Sepsis  25.5 Infective endocarditis  5.9 Pneumonia  5.9 Spontaneous bacterial peritonitis | IV Pen G+gentamicin  IV cloxacillin +gentamicin  IV Cefotaxime  IV Cetriazone and metronidazole | 17.6 Hearing loss:  - 11.8 Permanent deafness  - 5.8 Temporary hearing loss |
| Chau et. al. 1983 ^15^ | Hong Kong | Case series | 8 | 47.4 (24-71) | 87.5 | NR | 100 | NR | NR | 100 Meningitis¶  50 Arthitis | IV Penicillin G | 87.5 Hearing loss  Note: 1 patient had no data  1 Loss of balance |

| Study | Country | Study design | Sample size | Mean age (yr) (range) | Male (%) | Major risk factors % | | | | Main clinical presentations (%) | Treatment | Outcomes  (%) |
| --- | --- | --- | --- | --- | --- | --- | --- | --- | --- | --- | --- | --- |
|  |  |  |  |  |  | Recent exposure with pigs/porkª | Related occupation^b^ | Raw pork consumption^c^ | Others |  |  |  |
| Khadthasrima et. al. ^39^ | Thailand | Outbreak investigation report | 50  *Note:* 29 laboratory confirmed, 21 suspected cases | 49* (10-77) | 56 | NR | NR | NR  *Note:* All 9 cases interviewed reported consumption of raw pork | NR | 2 STSS  100 Fever  >80 Myalgia  60 Headache  >20 Nausea/ Vomiting  20 Diarrhea  10 Meningitis  10 Altered consciousness  < 10 Neck stiffness  5 Ecchymosis  <5 Seizure  <5 Arthralgia | NR | 96 Hospitalized  14 ICU (Intensive care Unit)  6 Death  5 Hearing loss |
| Thayawiwat et. al. 2012 ^40^ | Thailand | A public health surveillance study | 31 | 49.7 (21-70) | 80.6 | 3.2 | NR | 71 | 83.9 Alcohol drinking | 93.5 Fever  77.4 Headache  64.5 Myalgia  48.8 Neck stiffness  48.4 Nausea/  vomiting | NR | 51.6 Hearing loss |

ª History/recalled of exposure with pigs or pork before illness without slaughtering; ^b^ Related occupation includes farmer, butcher, abattoir worker, seller of raw pork; ^c^ Consumption of undercooked pork including raw pig blood, intestine and other internal organs; * Median age; ** Among 215 cases; 149 probable cases, 66 confirmed cases. A probable case referred to a compatible clinical illness without laboratory evidence. A confirmed case was defined as a compatible clinical illness with *S. suis* isolated verified from a normal sterile site despite the exposure; ^ PRRS: Porcine Reproductive and Respiratory Syndrome; ^^ Case series of Dutch and non-Dutch population. Non-Dutch data was excluded as the information was derived from literature; ¶ Study in bacterial meningitis patients; ¶¶ Classic triad signs of meningitis: fever defined as body temperature ≥ 38.5 Degree Celsius, neck stiffness, photophobia or a change in mental status defined as a Glasgow Coma Score < 14; NR: Not reported; ALF: Acute liver failure; ARF: Acute renal failure; ARDS: Acute Respiratory Distress Syndrome; DM: Diabetes Mellitus; DIC: Disseminated intravascular coagulation; HT: Hypertension; RHD: Rheumatic Heart Disease; STSS: Streptococcal Toxic Shock Syndrome; SBE: Subacute bacterial endocarditis

**Table S2:** Risk of bias assessment (RoB2.0): detailed notes for randomized, double-blind, placebo-controlled trial* ^10,51^

| **Domain** | **Signalling questions** | **Rating** | **Description/Support for judgement** |
| --- | --- | --- | --- |
| **1. Bias arising from the randomization process** | 1.1 Was the allocation sequence random? | Yes | Quote: "A computer-generated sequence of random numbers was used to assign treatment in blocks of 100 patients. If a patient met the entry criteria, the attending physician instructed a nurse to open a numbered envelope containing instructions to give either active drug or placebo".  P. 2432 (Mai, NT et. al. 2007)  Quote: "All patients, the physicians who enrolled them, and study investigators were unaware of the treatment assignments until the last patient had completed follow-up" P. 2432 (Mai, NT et. al. 2007) |
|  | 1.2 Was the allocation sequence concealed until participants were recruited and assigned to interventions? | Yes |  |
|  | 1.3 Were there baseline imbalances that suggest a problem with the randomization process? | No | Quote: "The study medication was given 15 minutes before the administration of antibiotics, although some patients may have had prior antibiotic treatment". P. 2432 (Mai, NT et. al. 2007) "There were no significant difference in the baseline characteristics between the study groups", P. 2435 (Mai, NT et. al. 2007) *Comment: Chance imbalances are not bias. |
|  | **Risk of bias judgement** |  |  |
|  | Optional: What is the predicted direction of bias arising from the randomization process? | Low |  |
| **2. Bias due to deviations from intended interventions** | 2.1. Were participants aware of their assigned intervention during the trial? | No | Quote: "All patients, the physicians who enrolled them, and study investigators were unaware of the treatment assignments until the last patient had completed follow-up" P. 2432 (Mai, NT et. al. 2007) |
|  | 2.2. Were carers and trial personnel aware of participants' assigned intervention during the trial? | No |  |
|  | 2.3. If Y/PY/NI to 2.1 or 2.2: Were there deviations from the intended intervention beyond what would be expected in usual practice? |  |  |
|  | 2.4. If Y/PY to 2.3: Were these deviations from intended intervention unbalanced between groups *and* likely to have affected the outcome? |  |  |
|  | 2.5 Were any participants analysed in a group different from the one to which they were assigned? | Yes | ITT approach was employed in the trial analysis.  Quote: Dexamethasone gr "2 were loss to FU, 1 discontinued dexamethasone"; placebo gr, 4 were loss to FU after 1 M, Figure 1 P. 2432 (Mai, NT et. al. 2007) Quote: "The study drug was withdrawn in one patient in each study group after 3 days because of bleeding in the upper gastrointestinal tract".  P. 2439 (Mai, NT et. al. 2007) |
|  | 2.6 If Y/PY/NI to 2.5: Was there potential for a substantial impact (on the estimated effect of intervention) of analysing participants in the wrong group? | PN | Comment: 76 received Dexamethaxone, 72 received placeo, 3 received neither. All patients initially received Cetriaxone 2 g q 12 h (Mai, NT., et al. 2008) |
| **Domain** | **Signalling questions** | **Rating** | **Description/Support for judgement** |
|  | **Risk of bias judgement** |  |  |
|  | Optional: What is the predicted direction of bias due to deviations from intended interventions? | Low |  |
| **3. Bias due to missing outcome data** | 3.1 Were outcome data available for all, or nearly all, participants randomized? | Yes | There was low number of loss to follow up according to Figure 1. Enrollment and Outcomes P. 2432 (Mai, NT et. al. 2007).  Outcomes were available in all 151 *S. suis* patients (Mai, NT., et al. 2008) |
|  | 3.2 If N/PN/NI to 3.1: Are the proportions of missing outcome data and reasons for missing outcome data similar across intervention groups? |  |  |
|  | 3.3 If N/PN/NI to 3.1: Is there evidence that results were robust to the presence of missing outcome data? |  |  |
|  | **Risk of bias judgement** |  |  |
|  | Optional: What is the predicted direction of bias due to missing outcome data? | Low |  |
| **4. Bias in measurement of the outcome** | 4.1 Were outcome assessors aware of the intervention received by study participants? | No |  |
|  | 4.2 If Y/PY/NI to 4.1: Was the assessment of the outcome likely to be influenced by knowledge of intervention received? |  |  |
|  | **Risk of bias judgement** |  |  |
|  | Optional: What is the predicted direction of bias due to measurement of the outcome? | Low |  |
| **5. Bias in selection of the reported result** | Are the reported outcome data likely to have been selected, on the basis of the results, from... |  |  |
|  | 5.1. ... multiple outcome measurements (e.g. scales, definitions, time points) within the outcome domain? | Yes |  |
|  | 5.2 ... multiple analyses of the data? | PN |  |
| **Risk of bias judgement** | Low / High / Some concerns | Low |  |
| Optional: What is the predicted direction of bias due to selection of the reported result? | Favours experimental / Favours comparator / Towards null /Away from null / Unpredictable | Favours experimental |  |
| **Overall bias** | **Risk of bias judgement** | Low |  |
|  | Optional: |  |  |

*The included article ^10^ does not contain information for all domains. Therefore, the information from the main study previously published ^51^ was also referred to for quality assessment.
